# A SMRT approach for targeted amplicon sequencing of museum specimens (Lepidoptera)—patterns of nucleotide misincorporation

**DOI:** 10.7717/peerj.10420

**Published:** 2021-01-14

**Authors:** Jacopo D’Ercole, Sean W.J. Prosser, Paul D.N. Hebert

**Affiliations:** 1 Centre for Biodiversity Genomics, Guelph, ON, Canada; 2 Department of Integrative Biology, University of Guelph, Guelph, ON, Canada

**Keywords:** Lepidoptera, HTS, Sequel, Museum specimens, COI, SMRT sequencing, Degraded DNA

## Abstract

Natural history collections are a valuable resource for molecular taxonomic studies and for examining patterns of evolutionary diversification, particularly in the case of rare or extinct species. However, the recovery of sequence information is often complicated by DNA degradation. This article describes use of the Sequel platform (Pacific Biosciences) to recover the 658 bp barcode region of the mitochondrial cytochrome *c* oxidase I (COI) gene from 380 butterflies with an average age of 50 years. Nested multiplex PCR was employed for library preparation to facilitate sequence recovery from extracts with low concentrations of highly degraded DNA. By employing circular consensus sequencing (CCS) of short amplicons (circa 150 bp), full-length barcodes could be assembled without a reference sequence, an important advance from earlier protocols which required reference sequences to guide contig assembly. The Sequel protocol recovered COI sequences (499 bp on average) from 318 of 380 specimens (84%), much higher than for Sanger sequencing (26%). Because each read derives from a single molecule, it was also possible to quantify the incidence of substitutions arising from DNA damage. In agreement with past work on sequence changes induced by DNA degradation, the transition C/G → T/A was the most prevalent category of change, but its rate of occurrence (4.58E−4) was so low that it did not impede the recovery of reliable sequences. Because the current protocol recovers COI sequence from most museum specimens, and because sequence fidelity is unaffected by nucleotide misincorporations, large-scale sequence characterization of museum specimens is feasible.

## Introduction

Although long valued for morphological studies, museum specimens are now also viewed as a rich, albeit largely untapped, genetic resource (Meineke & Davies, 2018). They not only provide the opportunity to assess genetic changes through time, but can be essential for species that are rare or extinct (Fleischer et al., 2006; Palkopoulou et al., 2018). Additionally, systematic and taxonomic studies often require the analysis of type specimens because they provide the only unambiguous link to the proper application of a Linnaean name when the presence of closely allied species complicates such designation (Yoshimoto, 1978). As a result, over the past decade, studies have aimed to recover sequences from museum specimens of plants (Nikolov et al., 2019), fungi (Trudell et al., 2017), and diverse lineages of animals including birds (Grealy, Bunce & Holleley, 2019), mammals (Schäffer, Zachos & Koblmüller, 2017), reptiles/amphibians (Chambers & Hebert, 2016), arthropods (Mitchell, 2015; Mikheyev et al., 2017), and various marine phyla (Jaksch et al., 2016). However, in many cases, DNA degradation limited the recovery of target sequences.

Sequences for the 658 bp barcode region of COI have helped to resolve taxonomic uncertainties (Speidel et al., 2015; Hausmann et al., 2016; Nazari et al., 2016), but it has often proven impossible to recover >200 bp amplicons from specimens older than 30 years old (Hebert et al., 2013; Allentoft et al., 2012). Sanger sequencing can characterize multiple short overlapping fragments to deliver a complete 658 bp sequence, but analytical costs are high (Hajibabaei et al., 2006; Hausmann et al., 2009; Lees et al., 2011; Hebert et al., 2013). In this situation, high-throughput sequencers (HTS) offer a major advantage because their capacity to characterize single molecules means they can deliver results with much lower concentrations of input DNA. Also, their ability to analyze amplicon pools generated through the use of several primer sets on DNA extracts from multiple specimens provides a cost-effective protocol for generating full-length barcodes (Prosser et al., 2016).

The first study using HTS to generate full-length DNA barcodes from museum specimens (Prosser et al., 2016) employed the Ion Torrent PGM platform (Thermo Fisher, Waltham, MA, USA). While sequences from this instrument rarely possess artifactual nucleotide substitutions, they are prone to indels, especially in homopolymer tracts. As a result, read depth must be at least 10X (S. Prosser, 2019, personal observations) to generate a reliable sequence for an amplicon. As well, the 3′ end of each read tends to be low quality so it is often trimmed during data processing. Because the extent of trimming is determined by the quality of each base call, reads for a particular amplicon typically vary in length (Fig. S1). In many cases, the resultant reads are so truncated that amplicons which should overlap fail to do so. This deficit can be overcome by assembling each read against a reference sequence from a closely allied species. In this way, truncated reads can be assembled into sequence with gaps in regions lacking coverage. While assemblies based on reference sequences are effective for taxa whose close relatives have full-length COI barcodes, contig assembly collapses when the nearest reference sequence is more than 10% divergent from the target (Table S1). This need for a close reference sequence acts as a barrier for taxa whose nearest neighbors have not been sequenced. Furthermore, the reference sequence approach can only be employed when the taxonomy of the target specimen is known (e.g., to a genus), and it constrains the extent to which data analysis can be automated. As a result, a method was needed that was not reliant on reference sequences.

The Sequel platform combines single-molecule real-time (SMRT) analysis with the capacity for circular consensus sequencing (CCS). This approach can generate high quality, full-length reads for multiple amplicons from each specimen, enabling the quantification of nucleotide substitutions resulting from in vivo and *post-mortem* degradation. Hydrolysis and oxidation are the main factors which modify the primary structure of DNA by fragmenting DNA strands, by creating oxidated nucleotides that hinder or block polymerase activity, and by inducing the incorporation of erroneous nucleotides during PCR (Pääbo, 1989; Höss et al., 1996). The first two types of damage compromise PCR amplification so no sequence is recovered, but the latter leads to substitutions that can, if frequent, lead to an invalid sequence. Because it is not possible to identify the strand on which damage first occurred, misincorporations are grouped into strand-complementary pairs. For instance, a C → T transition might reflect the modification of a C (prompting the observed transition), or the modification of a G on the complementary strand (producing a G → A transition). In a similar way, the 12 possible changes arising from nucleotide damage fall into six groups, two transitions (i.e., C/G → T/A, T/A → C/G) and four transversions (i.e., A/T → T/A, C/G → G/C, C/G → A/T, T/A → G/C). Although all six have been observed, the transition from C/G → T/A is most common (Pääbo, 1989; Lindahl, 1993). While some studies have only reported this transition (Hofreiter et al., 2001; Sawyer et al., 2012), others (Gilbert et al., 2003; Binladen et al., 2006) found a substantial incidence (24–30%) of the other transition (T/A → C/G). Although transversions are typically rare, Hansen et al. (2001) and Binladen et al. (2006) reported that the four transversions collectively represent about 20% of all substitutions induced by DNA damage.

The extent of post-mortem damage to mitochondrial DNA from museum specimens has rarely been examined (Sefc, Payne & Sorenson, 2007; Sawyer et al., 2012). However, SMRT sequencing makes it possible to accurately assess the extent of DNA degradation because high fidelity sequences are recovered, revealing variation which would have been overlooked with the earlier approach that required cloning PCR products.

## Materials and Methods

### Selection of museum specimens

Tissue sampling of 760 pinned butterflies was approved by the National Museum of Natural History (Washington). An effort was made to recover DNA barcodes from each specimen by amplifying two overlapping fragments (307 bp, 407 bp) of the COI barcode region followed by Sanger sequencing (Hajibabaei et al., 2006). About two thirds (485/760) failed to generate a barcode compliant sequence (>500 bp). From the group of failures, 380 specimens with an average age of 50 years and representing 110 species, 27 genera, and three families were selected for detailed comparison of sequence recovery with Sanger and Sequel analysis. Because prior studies on museum specimens using Sanger analysis have revealed marked differences in sequence recovery among similarly-aged specimens collected by different individuals (Hebert et al., 2013), the success of barcode recovery for sequences obtained with the Sequel was compared among specimens from the five collectors who contributed the most specimens.

### DNA extraction

One leg from each specimen was placed into 96-well Eppendorf plates (95 legs per plate plus one negative control). Fifty microliters of lysis buffer (30 mM Tris-HCl with pH 8.0, 700 mM guanidine thiocyanate, 30 mM EDTA with pH 8.0, 0.5% Triton X-100, 5% Tween-20, 2 mg/ml proteinase K) was added to each well and incubated at 56 °C for 18 h. Following incubation, a silica membrane-based approach was used to purify the DNA (for details see Ivanova, DeWaard & Hebert (2006)). Briefly, 100 µL of Binding Mix (5 mM Tris-HCl at pH 6.4, 3 M guanidine thiocyanate, 10 mM EDTA with pH 8.0, 2% Triton X-100, 50% ethanol) was added to each lysate and the 150 µL was transferred to a silica membrane plate (PALL Corporation). The membrane was washed with 180 µL of Protein Wash Buffer (2.6 mM Tris-HCl with pH 6.4, 1.56 M guanidine thiocyanate, 5.2 mM EDTA with pH 8.0, 1.04% Triton X-100, 70% ethanol) and 700 µL of Wash Buffer (10 mM Tris-HCl pH 7.4, 50 mM NaCl, 0.5 mM EDTA pH 8.0, 60% ethanol). The membrane was dried and DNA was eluted into a clean 96-well plate with 40 µL of Elution Buffer (10 mM Tris-HCl, pH 8).

### Amplification of short barcode fragments

The general workflow, involving nested PCR and primer multiplexing, followed Prosser et al. (2016) with modifications for SMRT sequencing. All reactions were performed in 96-well plates unless otherwise stated. Three rounds of PCR were required to (i) produce a spectrum of COI amplicons from each DNA extract, (ii) generate short, overlapping amplicons flanked by PacBio “PB1” adapters, and (iii) add unique molecular identifiers (UMIs) to the amplicons from each specimen so multiple samples could be pooled for sequencing.

The first round of PCR involved two reactions per sample (PCR1.1, PCR1.2). Reaction components followed Prosser et al. (2016). Each reaction contained three forward primers spanning the barcode region and 5–6 reverse primers (see Fig 1A in Prosser et al., 2016). This primer configuration generated up to 12 possible amplicons depending on the extent of DNA degradation and which primers successfully annealed. By strategically splitting the primers used in the first round of PCR into two reactions (PCR1.1, PCR 1.2), it was possible to avoid preferentially amplifying short overlap regions. This reaction employed untagged primers as primers with adapter/UMI tails generated far lower PCR products than untagged primers (Prosser et al., 2016). This difference is likely due to the formation of secondary structures that hinder polymerase activity, a problem that gains importance when template concentrations are low. The PCR regime consisted of 94 °C for 2 min, 60 cycles of 94 °C for 40 s, 48 °C for 40 s, and 72 °C for 30 s, and a final extension of 72 °C for 5 min.

The amplicons generated by the first round of PCR were pooled and size selected (>100 bp) via carboxylate-coated magnetic beads (SpeedBeads; Sigma Aldrich, St. Louis, MO, USA). A single vial of Speadbeads ($734 CAD) allows the purification of 50,000 PCR reactions, enough to process 12,500 specimens ($0.06 CAD each). Briefly, the entire PCR reaction (12.5 µL) was mixed with 14.4 µL of magnetic beads and incubated for 10 min at room temperature. The beads were immobilized on a magnet and washed three times with 120 µL of 80% ethanol. The washed beads were then dried before the amplicons were eluted with 30 µL of water for use in PCR2. The second round of PCR consisted of six reactions (PCR2.1, PCR2.2, PCR2.3, PCR2.4, PCR2.5, PCR2.6). Three (PCR2.1, PCR2.3, PCR2.5) used PCR1.1 as template, while the others (PCR2.2, PCR2.4, PCR2.6) used PCR1.2 as template. Reaction components were the same as those employed for the first round except the primers were tailed with PB1 adapters, which provided universal primer binding sites for subsequent fusion of the UMIs. Since the second PCR employs amplicons from the first PCR as template, primers bind perfectly, enabling uniform enrichment of the template molecules while adding the sequencing adapters. While UMIs could be added at this stage, this would require 1152 different primers for each plate (i.e., each of the 96 samples would require 6 forward and 6 reverse primers, each uniquely tagged). By comparison, the addition of universal primer binding sites (i.e., adapters), it only required 192 different primers for the third round of PCR. Each reaction combined one forward primer with two reverse primers, leading to the generation of ~150 bp and/or ~230 bp amplicons depending on which reverse primer paired with the forward primer. The ~150 bp amplicons are henceforth referred to as “singleton” while the ~230 bp are termed “duplex”. The inclusion of two reverse primers provided redundancy in cases where one reverse primer failed to bind. Furthermore, because duplex amplicons span the same barcode region as two singleton amplicons, they added redundancy if the forward primer for a particular singleton reaction failed to bind. Thermocycling conditions followed those for PCR1, but they were reduced to 40 cycles. Following thermocycling, all six PCR reactions were pooled for each sample, and a 12.5 µL aliquot of each pool was purified as above.

The purified products were then used for a third round of PCR which added UMI tags to the amplicons recovered from each specimen. In order to multiplex 96 samples in each sequencing run, asymmetrical dual-tagging was employed because 80% of reads labeled with two unique tags could be assigned to their source specimen vs 60% with a single tag. Consequently, the third round of PCR required 96 different forward primers and 96 different reverse primers. The UMI-tagged fusion primers were complementary to the PB1 adapters of the PCR2 primers. Thermocycling consisted of 94 °C for 2 min, 20 cycles of 94 °C for 40 s, 64 °C for 40 s, and 72 °C for 1 min, followed by final extension of 72 °C for 5 min. After thermocycling, the amplicons contained sample-specific UMI tags, allowing them to be pooled for sequencing.

### Preparation of amplicons for SMRT sequencing

PacBio instructions for amplicon sequencing were followed to prepare libraries for SMRT sequencing. Briefly, 400 µL of the library was purified using 480 µL of AMPure-PB beads (1.2X). All subsequent purifications were carried out using the same 1.2X beads-sample ratio. End-repair and SMRTbell adapter ligation followed PacBio recommendations; the latter reaction was performed at room temperature for 1 h. Primer annealing and polymerase binding were also performed following PacBio’s recommendations and the polymerase-bound products were loaded onto a SMRT cell (1M v2) via diffusion loading without prior enrichment at a concentration of 18 pM.

### SMRT sequencing and generation of circular consensus sequences

Sequencing run parameters were set using SMRTLink version 5.0 and sequencing was performed on a Sequel platform. Default run settings were used with the following exceptions: insert size was set to 500, movie time to 480 min, immobilization time to 120 min, and pre-extension time to 20 min. Following sequencing, the raw data was analyzed using the CCS algorithm under the SMRT Analysis module of SMRTLink. Default settings were used with the following exceptions: the maximum and minimum subread lengths were set to 500 bp and 100 bp respectively.

### De novo assembly of CCS reads

CCS reads were downloaded in FASTA format and run through custom bash and R scripts (Data S1–S7) that processed the reads and assembled them into full-length barcode contigs. All analyses employed standard Linux bash commands unless otherwise noted. First, the 5 bp pad sequence was trimmed from both ends of each read using CutAdapt (Martin, 2011). Next, the reads were demultiplexed using the fastx barcode splitter from the Fastx Toolkit (http://hannonlab.cshl.edu/fastx_toolkit/index.html). Each read was then assigned to its source sample by examining its forward and reverse UMI tags. For a UMI tag to be identified, it had to perfectly match one of the 192 UMI tags (96 forward, 96 reverse) used for amplicon labeling, but only one of the two UMI tags on a sequence needed to meet this criterion for its attribution to a specimen. Following demultiplexing, each read was compared via BLAST to all available unique BOLD BINs (Ratnasingham & Hebert, 2013) (boldsystems.org) (Ratnasingham & Hebert, 2007) (as of April 2018). The purpose of this BLAST search was to identify the taxonomic source of each read at an ordinal level. The ordinal level identifications were then compared to that for each source organism, and rare cases of mismatches were removed from the dataset. As all DNA extracts in this study derived from Lepidoptera, reads showing closest similarity to another insect order were excluded from further analysis.

**Figure 1:**
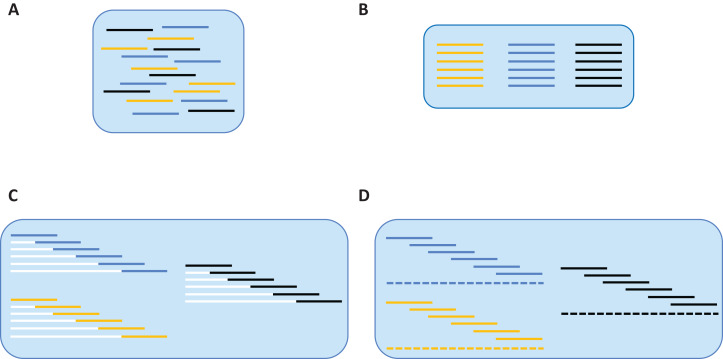
De novo assembly of SMRT reads to produce a contiguous barcode sequence. (A) Reads from different samples are associated with their source specimen via UMI tags. (B) Within each sample, reads are associated with their source fragment of COI via forward primer sequences. (C) Based on the relative position of each amplicon within the barcode region, a specific number of non-IUPAC characters is inserted upstream of the sequence, forcing it into alignment. (D) A contig (barcode sequence) is generated via majority consensus of the aligned reads.

Reads that passed this taxonomy filter were employed for de novo assembly which was performed iteratively for all samples (Fig. 1). Reads from a particular sample were identified via their UMIs (Fig. 1A), and they were then partitioned based on their forward primer (i.e., one of six), or failing that, their reverse primer (i.e., one of six) (Fig. 1B). Reads that could not be partitioned by either primer were discarded. As the position of each primer within the barcode region is known, the relative position of each read was certain. Each read was then forced into its relative position by appending a specific number of non-IUPAC characters to its 5′ end (white lines of Fig. 1C). For example, a read that was supposed to start at nucleotide 145 would be assigned 144 non-IUPAC characters. Once all reads were forced into relative alignment, a consensus sequence was generated for each fragment to remove sequence variation linked to polymerase errors. When a majority consensus could not be reached at a position, a N was registered in the consensus sequence. If this process led to more than two Ns in the sequence, it was excluded from further analysis. The consensus sequence for each fragment was then used to create a contig spanning the barcode region (dashed line of Fig. 1D). However, before generating the final contig, each fragment’s consensus sequence was replicated to reflect the number of reads from which it was derived. This was done to add weight to the consensus of each fragment so the final contig sequence accurately reflected the majority of the reads in rare cases of discordance between overlapping reads. As the non-IUPAC characters were ignored, the final barcode sequence was based solely on real data. If a segment of the barcode region lacked coverage, but was flanked by areas with sequence data, the gap was filled with Ns so only a single sequence was produced per sample. Once a sequence was assembled for each of the 95 samples in a plate, the sequences were combined into a single FASTA file to aid downstream validation.

### Sequence validation

The output of the de novo assembly consisted of one “assembly” FASTA file as well as a single “master” FASTA file per sample. The “assembly” file contained the component reads resulting from each sample and their consensus sequence (i.e., the final barcode sequence). The “master” FASTA file contained consensus sequences (i.e., final barcode sequences) for each of the samples included in the run. Prior to upload to BOLD, the “master” FASTA file was manually examined in AliView (Larsson, 2014) for indels and for evidence of contamination events or chimeras resulting from the inclusion of non-target sequences in the assembly. The sequences were also checked for stop codons via amino acid translation. The “assembly” FASTA files for sequences failing one or more of these checks were examined to determine the cause of the issue. Conflicting reads were identified via the Identification Engine on BOLD and the assembly was corrected. If conflicting reads could not be resolved, the affected region was masked with Ns in the final consensus sequence. Sequences of the “master” file were further analyzed using a Neighbor-Joining tree and barring unexpected phylogenetic placements, they were uploaded to BOLD. These measures ensured that only reliable sequences were uploaded to BOLD.

### Characterization of sequence changes induced by post-mortem damage

Prior studies have linked heterogeneity in sequences recovered from subfossils to PCR errors and post-mortem damage (Pääbo, Higuchi & Wilson, 1989), while only PCR errors contribute to sequence variation in recently collected specimens (Dunning, Talmud & Humphries, 1988). Based on this difference, the extent of intra-individual variation was compared between sequences recovered from five old museum specimens (average age = 69 years) and from five newly collected specimens (Table 1) to quantify the extent of sequence variation linked to post-mortem damage. The error rates were normalized with respect to GC composition across the alignment. The fresh samples were collected in 2017–2018 and were held in 95% ethanol at −20 °C until DNA extraction. All 10 specimens were processed using the same PCR and sequencing protocols. The data were filtered to retain only CCS reads with a minimum estimated sequencing accuracy of 99.99%. Geneious ver. 11.1.5 (https://www.geneious.com) was employed to assemble the reads and to generate the consensus for the full barcode sequence. The consensus sequence was then employed as a reference to assess sequence diversity among the CCS reads. Among the various possible categories of sequence variation, only SNPs were retained as they can reflect errors during PCR or degradation of the DNA molecules. All reads were compared against the consensus and those diverging more than 3% were removed on the presumption that they represented contaminants or NUMTs. CCS reads with stop codons were also discarded as they likely represent NUMTs. Identical substitutions at a particular site in more than one CCS read from a particular specimen were regarded as reflecting a single event regardless of their frequency. Although this approach will underestimate error rates if a particular sequence change occurred in multiple amplicons, it was presumed that the same substitution is unlikely to happen more than once at a site. Mann–Whitney tests, one for each of the six categories of substitution (confidence interval = 95%, 4 degrees of freedom), were employed to compare substitution rates between museum and fresh samples. All reads were characterized based on the L-strand.

**Table 1:** Samples employed to assess substitutions induced by post-mortem damage.

Process ID	Species	Collection year	Age (years)	Collection locality	Institution storing
NGSFT3956-16	*Euchloe lotta*	1955	61	USA, Washington	NMNH
NGSFT3963-16	*Eurema daira*	1938	78	USA, Georgia	NMNH
NGSFT3869-16	*Heliopetes laviana*	1944	72	USA, Texas	NMNH
NGSFT3828-16	*Erynnis meridianus*	1958	58	USA, Arizona	NMNH
NGSFT3929-16	*Amblyscirtes vialis*	1941	75	USA, Virginia	NMNH
JSPEC127-18*	*Nymphalis antiopa*	2017	1	Canada, Ontario	CBG
JSPEC123-18*	*Cercyonis pegala*	2018	0	Canada, Ontario	CBG
JSPEC120-18*	*Glaucopsyche lygdamus*	2018	0	Canada, Ontario	CBG
JSPEC074-18*	*Limenitis arthemis*	2017	1	Canada, Ontario	CBG
JSPEC128-18*	*Pieris rapae*	2018	0	Canada, Ontario	CBG

**Note:**

Asterisks indicate fresh samples; the last two digits of the Process ID indicate the year when each sample was processed (e.g., 16 stands for 2016); NMNH refers to National Museum of Natural History; CBG refers to Center for Biodiversity Genomics.

## Results

### Methodological efficacy

**Figure 2:**
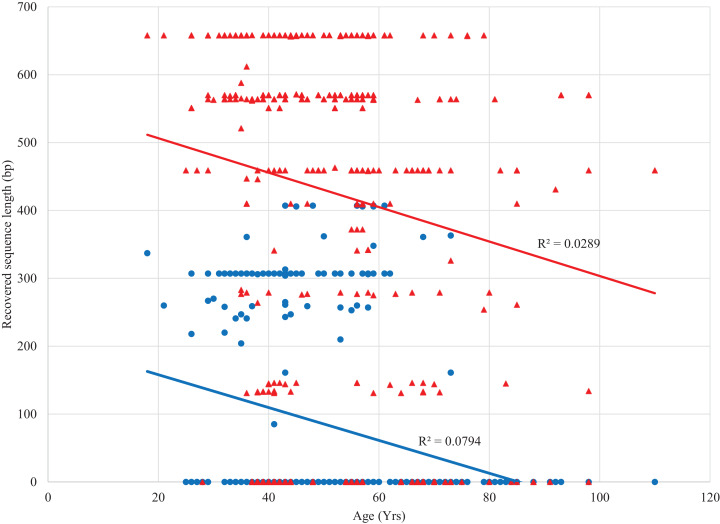
Relationship between length of the COI sequence recovered and age of the source specimen with Sanger (blue) and SMRT sequencing (red). Trend lines and *R*^2^ values are shown.

**Figure 3:**
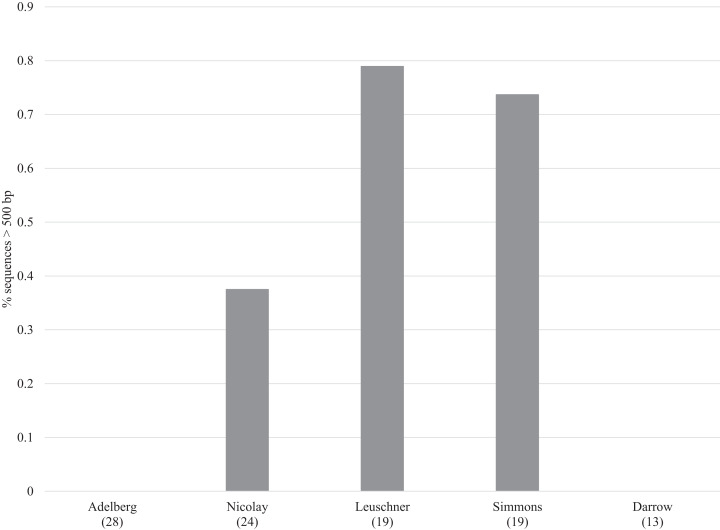
Recovery of barcode compliant sequences (i.e., >500 bp) for specimens from five collectors. The number of samples from each collector is in brackets.

Sanger sequencing of 307/407 bp amplicons from the 380 specimens yielded sequences from 100 specimens (26%) with an average length of 298 bp (range: 87–407 bp) (dx.doi.org/10.5883/DS-LEPSAN, Fig. 2). By comparison, SMRT analysis recovered sequences averaging 499 bp (range: 131–658 bp) from 318 specimens (84%), including all that yielded Sanger data. Among these specimens, 23% were full-length (658 bp), 50% were >500 bp, and 69% were >300 bp (dx.doi.org/10.5883/DS-LEPSEQ, Fig. 2). Sequence recovery across the 658 bp barcode region varied. Nucleotide positions 1–326 and 526–658 were recovered with high success (74% each), but positions 327–525 were recalcitrant (40%). Success in barcode recovery also varied among specimens with different collectors (Fig. 3). Three quarters of the 37 specimens from Leuschner and Simmons delivered >500 bp sequences, while none of the 41 from Adelberg and Darrow reached 460 bp. The 24 specimens collected by Nicolay showed intermediate success with 38% yielding sequences >500 bp.

### Sequence heterogeneity in fresh and museum specimens

All ten samples (Table 1) delivered 658 bp sequences and all base calls were unambiguous. Rates of substitution (Figs. S2–S12; Table S2) were higher in museum than fresh specimens for each category of transition C/G → T/A (*p* < 0.01); T/A → C/G (*p* = 0.04) and transversion (A/T → T/A (*p* = 0.02); C/G → G/C (*p* = 0.07); C/G → A/T (*p* = 0.03); T/A → G/C (*p* = 0.1)) (Table S3).

**Figure 4:**
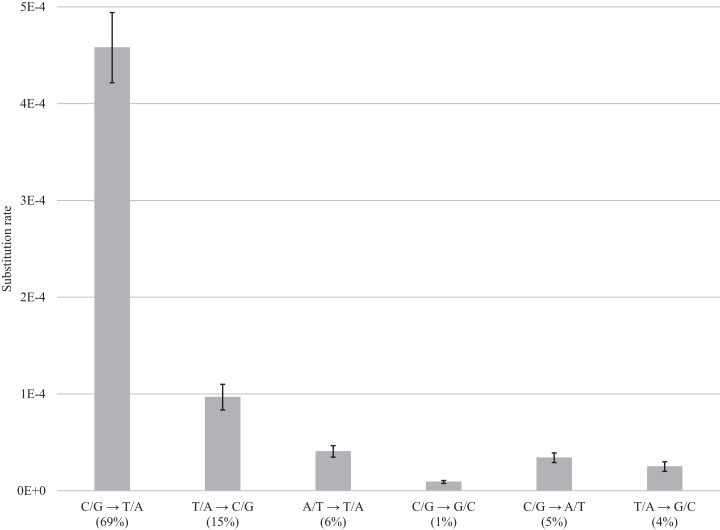
DNA decay induced errors. Substitution rates for each type of transition and transversion are obtained by subtracting rates for fresh samples from the rates for museum samples; bars show the standard error. Values in brackets refer to the percentage of errors for a particular category of substitution. ****

After normalization for GC composition, the C/G → T/A transition was most frequent in museum specimens, occurring at a frequency of 4.58E−4 (69% of the total). The other changes were less common ranging from 9.67E−5 (15%) for T/A → C/G to 4.06E−5 (6%) for A/T → T/A, 9.04E−06 for C/G → G/C (1%), 3.41E−5 for C/G → A/T (5%), and 2.5E−5 for T/A → G/C (4%) (Fig. 4; Table S2). Summing the incidence of all six categories revealed a total frequency of 6.63E−4 (Table S2).

## Discussion

### SMRT sequencing of degraded DNA

Single-molecule real-time analysis increased sequence recovery threefold while sequence length was nearly doubled, when compared to Sanger. Moreover, analytical costs were substantially reduced (Hebert et al., 2018). While the current protocol was developed for use on short-read HTS platforms, its deployment on the long-read Sequel enabled contig assembly without a reference sequence. This was possible because the forward/reverse primer sequences in each CCS revealed its position in the contig, allowing the automated generation of a consensus sequence. In cases where recovery of the 658 bp amplicon was incomplete, gaps denoted by Ns could produce a full-length contig, incorporating all available information into a single sequence. Transition to Sequel brought a second advantage as it improved data retention by enabling dual-UMI tagging.

Most museum specimens (84%) analyzed with the Sequel platform produced a sequence, but less than 50% yielded coverage for nucleotide positions 327–525. The primer binding sites in this region of COI have high variability within Lepidoptera, leading to primer-template mismatches. Although primer degeneracy was employed to alleviate this problem, amplification success could be further increased by employing taxon-specific primer sets, but this would limit the general application of the protocol.

Migration of the protocol of Prosser et al. (2016) from ion semiconductor sequencing to SMRT sequencing was primarily motivated to enable de novo read assembly, a process that requires high quality, full-length reads. While the in-house Sequel platform was utilized in the present study, a similar approach could be employed on the Sequel II; its increased read output potentially allows the analysis of 8X as many specimens in a run, reducing costs (Data S8; $375 CAD per million reads). A similar protocol might also be deployed on the Illumina MiSeq platform. In this case, use of either the 2 × 250 bp or 2 × 300 bp chemistry should enable recovery of the longest amplicons (ca. 350 bp), and de novo assembly of merged paired-end reads should be possible using the scripts employed in the current analysis. The same chemistry could also be used on the high-end Illumina NovaSeq, but its adoption sequencer would only require a portion of each Flow Cell. So long as target fragments are short (<350 bp), Illumina platforms can undoubtedly recover sequences from museum specimens at lower cost than on Sequel II (Data S8).

Hebert et al. (2013) reported marked variation in the success of sequence recovery from museum specimens processed by different collectors, and the same pattern was detected in this study. Although the differing treatments responsible for this variation are uncertain, killing agents and preservation methods are known to impact DNA degradation (Mandrioli, Borsatti & Mola, 2006; Dean & Ballard, 2001). The strength of these impacts makes clear the importance of screening subsets of museum samples before initiating any extensive sequencing project.

### Examination of DNA damage through SMRT sequencing

Prior efforts to characterize DNA damage have largely examined fossil or subfossil samples. By contrast, the present study quantified the incidence of nucleotide misincorporations in museum samples. It is first important to emphasize that multiple factors can create variation in the sequences recovered from a specimen. Contamination during analysis, environmental DNA (eDNA) associated with the specimen, and NUMTS can all lead to the recovery of very divergent sequences. Aside from sequence variation introduced by these factors, PCR errors and variation in the number of template molecules can influence the extent of heterogeneity among sequences recovered from the actual specimen. To exclude variation from contamination, eDNA or NUMTS, we removed reads with >3% divergence from the consensus sequence on the basis that they likely reflected non-target amplification. Secondly, to quantify PCR errors, we examined sequence variation among reads from fresh specimens. This analysis revealed a substitution rate (8.8E−4) within the predicted range (6.0E−4–1.2E−2) based on fidelity of the Taq polymerase employed and the number of PCR cycles (Eckert & Kunkel, 1991) (Table S2). Because this error rate is so low, PCR error has negligible impact on the final consensus sequence because of the high read coverage obtained for each specimen (range: 165X to 517X). Thirdly, variation in the number of template molecules can influence the extent of variation among the sequences from different specimens (Reiss et al., 1990). For instance, if only a single template molecule is present, all PCR products would be identical, and the resulting amplicons would lack variation linked to nucleotide damage. Conversely, if many template molecules with minor sequence differences are amplified, the amplicons would reflect this variation. In the present study, variation in template numbers did not seem to impact results because substitution rates were similar among the five museum specimens.

In agreement with prior studies on the decay of the primary structure of DNA in vivo (Lindahl, 1993), in fossils (Höss et al., 1996), and in museum samples (Sefc, Payne & Sorenson, 2007; Sawyer et al., 2012), the transition C/G → T/A represented the predominant substitution (69%) detected in this study. Because our protocol employed PCR prior to sequencing, it is impossible to determine if the mutation in the original template DNA was C → T or G → A. However, Hofreiter et al. (2001) noted that G → A is unlikely from a biochemical perspective, while the well-known pathway leading to the deamination of cytosine to uracil (a thymine analog that binds adenine) makes it the probable mechanism provoking this transition. The second commonest substitution in this study was the transition T/A → C/G which accounted for 15% of all changes. Some prior studies failed to detect this substitution (Hofreiter et al., 2001; Stiller et al., 2006), but others found that it comprised 30% of all changes (Binladen et al., 2006; Gilbert et al., 2003). Results need to be interpreted with caution because the transition T/A → C/G represents the commonest PCR error (McInerney, Adams & Hadi, 2014) so some may persist in our dataset and in other published data explaining the variable outcomes. Lastly, although transversions are typically less frequent than transitions, they accounted for about 20% of all changes in some studies (Hansen et al., 2001; Binladen et al., 2006). Our work confirmed this result, showing that the four transversions contribute 16% of all changes, with A/T → T/A being the most frequent (6%). Similar to the transition T/A → C/G, this transversion A/T → T/A represents a characteristic PCR error with Taq polymerase (Eckert & Kunkel, 1991). It is therefore again possible that undetected PCR errors have contributed to the observed rates for this transversion.

Because studies have targeted different organisms, gene regions, and have employed different PCR conditions and sequencing platforms, the comparison of absolute error rates across studies is not meaningful. However, it remains critical to estimate errors associated with DNA damage as they could affect the validity of the barcode sequences recovered from museum specimens. Our analysis revealed that the most common substitution occurred at a frequency of 4.58E−4 per bp (ca. one error per 17 reads), while the total for all six types of substitutions is 6.63E−4 per bp (ca. one error per 12 reads) (Table S2). Because the Sequel platform generated an average read coverage per specimen varying from 165X to 517X, it is expected that the consensus sequences generated in this study were unaffected by substitutions linked to DNA degradation.

## Conclusion

This study employed circular consensus sequencing on Sequel to characterize short amplicons (ca. 150 bp) generated by nested PCR of DNA extracts derived from old museum specimens of Lepidoptera. Sequence recovery was high with 50% of the specimens generating at least 500 bp coverage for the 658 bp amplicon. In contrast to prior protocols which required a reference sequence for contig assembly, the Sequel protocol does not, allowing for automated contig assembly. The analysis of sequence variation among amplicons from fresh and museum specimens revealed an elevated rate of substitutions in museum specimens, reflecting *post-mortem* degradation. However, the incidence of these changes was too low to impede the recovery of valid sequences. Based on these results, the coupling of nested PCR with sequence characterization on Sequel provides an effective workflow for recovering sequence information from museum specimens.

## Supplemental Information

10.7717/peerj.10420/supp-1 Supplemental Information 1 Effect of increased genetic distance between reads and reference sequences on reference-based contig assembly. The same set of sequence reads were aligned to various reference sequences ranging from closely to distantly related. The number of aligned reads and the resulting number of aligned base pairs are shown for each reference sequence.

10.7717/peerj.10420/supp-2 Supplemental Information 2 Substitution rates for six types of substitution. Asterisks indicate fresh samples. Rates in column I and row 16 are normalized with respect to GC composition. The total expected substitution rate was calculated following Eckert & Kunkel (1991) where coverage refers to the arithmetic mean of the read coverage for the six fragments spanning the barcode region. Errors induced by DNA decay were quantified by subtracting the substitution rate for fresh samples from the rate for museum samples. Values in brackets refer to the percentage of errors for a particular category of substitution.

10.7717/peerj.10420/supp-3 Supplemental Information 3 Mann-Whitney test for substitution rates grouped by type of substitution. Rates for museum samples were compared against rates for fresh samples by category of substitution.

10.7717/peerj.10420/supp-4 Supplemental Information 4 Examples of aligned Ion Torrent and SMRT reads. (a) Aligned Ion Torrent reads showing variable lengths following unidirectional sequencing and quality trimming. (b) Aligned SMRT reads showing near-constant lengths following circular consensus sequencing.

10.7717/peerj.10420/supp-5 Supplemental Information 5 Substitution rates for the museum specimen of *Euchloe lotta* (process ID: NGSFT3956-16).

10.7717/peerj.10420/supp-6 Supplemental Information 6 Substitution rates for the museum specimen of *Eurema daira* (process ID: NGSFT3963-16).

10.7717/peerj.10420/supp-7 Supplemental Information 7 Substitution rates for the museum specimen of *Heliopetes laviana* (process ID: NGSFT3869-16).

10.7717/peerj.10420/supp-8 Supplemental Information 8 Substitution rates for the museum specimen of *Erynnis meridianus* (process ID: NGSFT3828-16).

10.7717/peerj.10420/supp-9 Supplemental Information 9 Substitution rates for the museum specimen of *Amblyscirtes vialis* (process ID: NGSFT3929-16).

10.7717/peerj.10420/supp-10 Supplemental Information 10 Substitution rates for the fresh specimen of *Nymphalis antiopa* (process ID: JSPEC127-18).

10.7717/peerj.10420/supp-11 Supplemental Information 11 Substitution rates for the fresh specimen of *Cercyonis pegala* (process ID: JSPEC123-18).

10.7717/peerj.10420/supp-12 Supplemental Information 12 Substitution rates for the fresh specimen of *Glaucopsyche lygdamus* (process ID: JSPEC120-18).

10.7717/peerj.10420/supp-13 Supplemental Information 13 Substitution rates for the fresh specimen of *Limenitis arthemis* (process ID: JSPEC074-18).

10.7717/peerj.10420/supp-14 Supplemental Information 14 Substitution rates for the fresh specimen of *Pieris rapae* (process ID: JSPEC128-18).

10.7717/peerj.10420/supp-15 Supplemental Information 15 Average substitution rates for five museum (58-78 years old) and five fresh (0-1 years old) butterfly specimens. Substitution rates for each type of transition and transversion; bars show the standard error.

10.7717/peerj.10420/supp-16 Supplemental Information 16 Main Bash script. It was employed to trim, filter, demultiplex, and run the raw reads through the R scripts (see Data S6–S7).

10.7717/peerj.10420/supp-17 Supplemental Information 17 5’ PacBio forward UMIs. List of forward UMIs as they appear at the 5’ end of reads; it was used for demultiplexing by the main bash script.

10.7717/peerj.10420/supp-18 Supplemental Information 18 3’ PacBio forward UMIs. List of forward UMIs as they appear at the 3’ end of reads; it was used for demultiplexing by the main bash script.

10.7717/peerj.10420/supp-19 Supplemental Information 19 5’ PacBio reverse UMIs. List of reverse UMIs as they appear at the 5’ end of reads; it was used for demultiplexing by the main bash script.

10.7717/peerj.10420/supp-20 Supplemental Information 20 3’ PacBio reverse UMIs. List of reverse UMIs as they appear at the 3’ end of reads; it was used for demultiplexing by the main bash script.

10.7717/peerj.10420/supp-21 Supplemental Information 21 R script—filtering. It was employed to filter reads whose closest match was not Lepidoptera.

10.7717/peerj.10420/supp-22 Supplemental Information 22 R script— de novo assembly. It was employed for de novo assembly of reads that passed the taxonomy filter.

10.7717/peerj.10420/supp-23 Supplemental Information 23 Comparison of sequencing costs.

## Additional Information and Declarations

### Field Study Permissions

The following information was supplied relating to field study approvals (i.e., approving body and any reference numbers): Tissue sampling of museum samples was approved by the National Museum of Natural History (Washington).

### DNA Deposition

The following information was supplied regarding the deposition of DNA sequences: Raw HTS data are available at the Sequence Read Archive: SAMN14482007–SAMN14482391. Final barcode sequences, including specimen metadata for the 380 museum samples retrieved through Sequel and Sanger are available respectively under the BOLD dataset “DS-LEPSEQ” (DOI 10.5883/DS-LEPSEQ) and “DSLEPSAN” (DOI 10.5883/DS-LEPSAN). Final barcode sequences for the five freshly collected specimens are available under the BOLD dataset “DS-LEPSEQFR” (DOI 10.5883/DS-LEPSEQFR).

### Data Availability

The following information was supplied regarding data availability: The specimens described in the study are located at the National Museum of Natural History (Washington). Accession numbers for each specimen (Museum IDs) are available under the BOLD dataset “DS-LEPSEQ” (DOI 10.5883/DS-LEPSEQ), “DSLEPSAN” (DOI 10.5883/DS-LEPSAN), and “DS-LEPSEQFR” (DOI 10.5883/DS-LEPSEQFR). The Main Bash script employed to trim, filter, demultiplex, and run the raw reads through the R scripts, the R script employed to filter reads whose closest match was not Lepidoptera and the R script employed for de novo assembly of reads that passed the taxonomy filter are available as Supplemental Files.
