# Supplementary figures and images for: A SMRT approach for targeted amplicon sequencing of museum specimens (Lepidoptera)—patterns of nucleotide misincorporation
Source: PeerJ. 2021 Jan 14;9:e10420. doi: 10.7717/peerj.10420 (PMC7811786; doi:10.7717/peerj.10420)

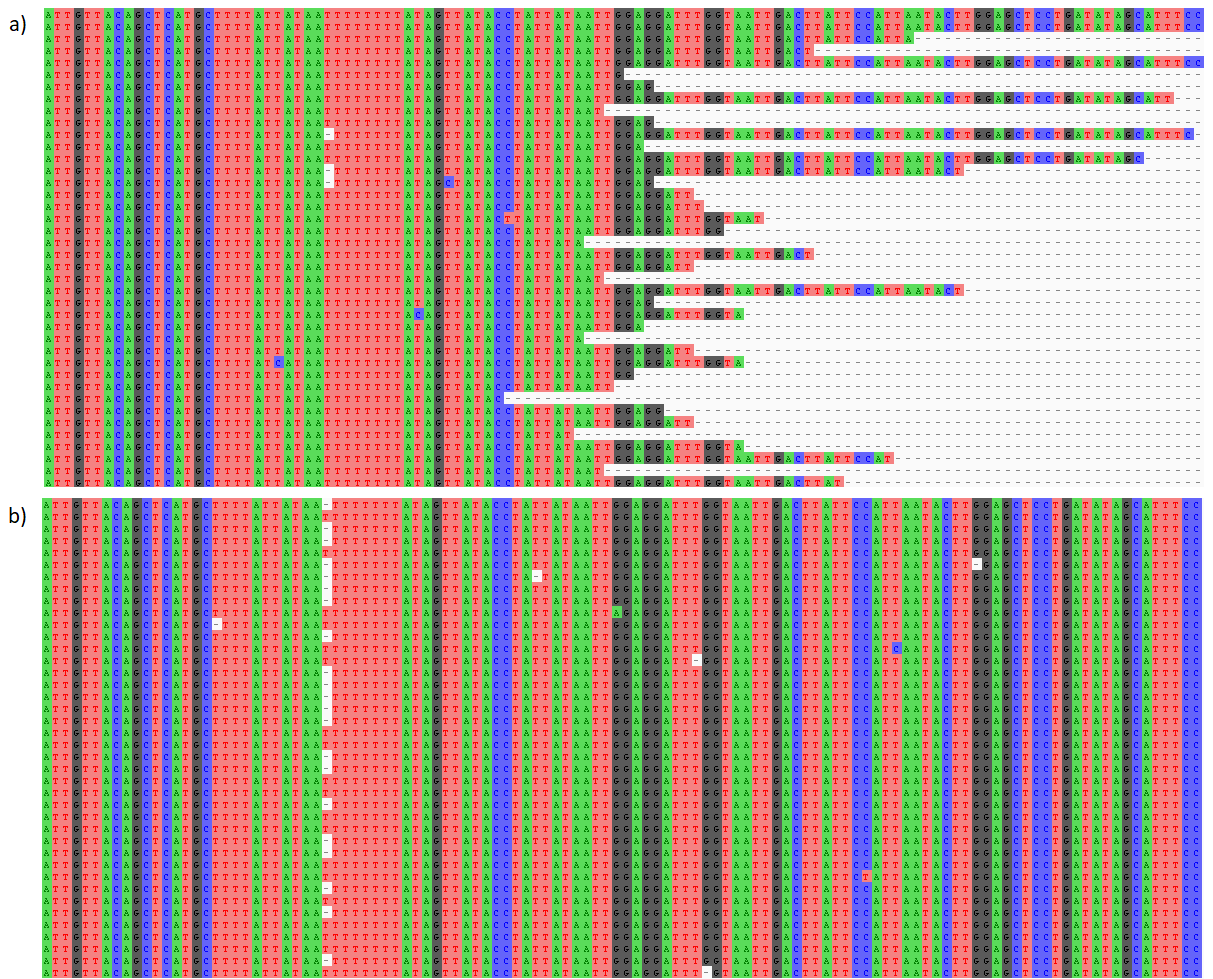

Supplement: Supplemental Information 4 — (a) Aligned Ion Torrent reads showing variable lengths following unidirectional sequencing and quality trimming. (b) Aligned SMRT reads showing near-constant lengths following circular consensus sequencing. [file peerj-09-10420-s004.png]

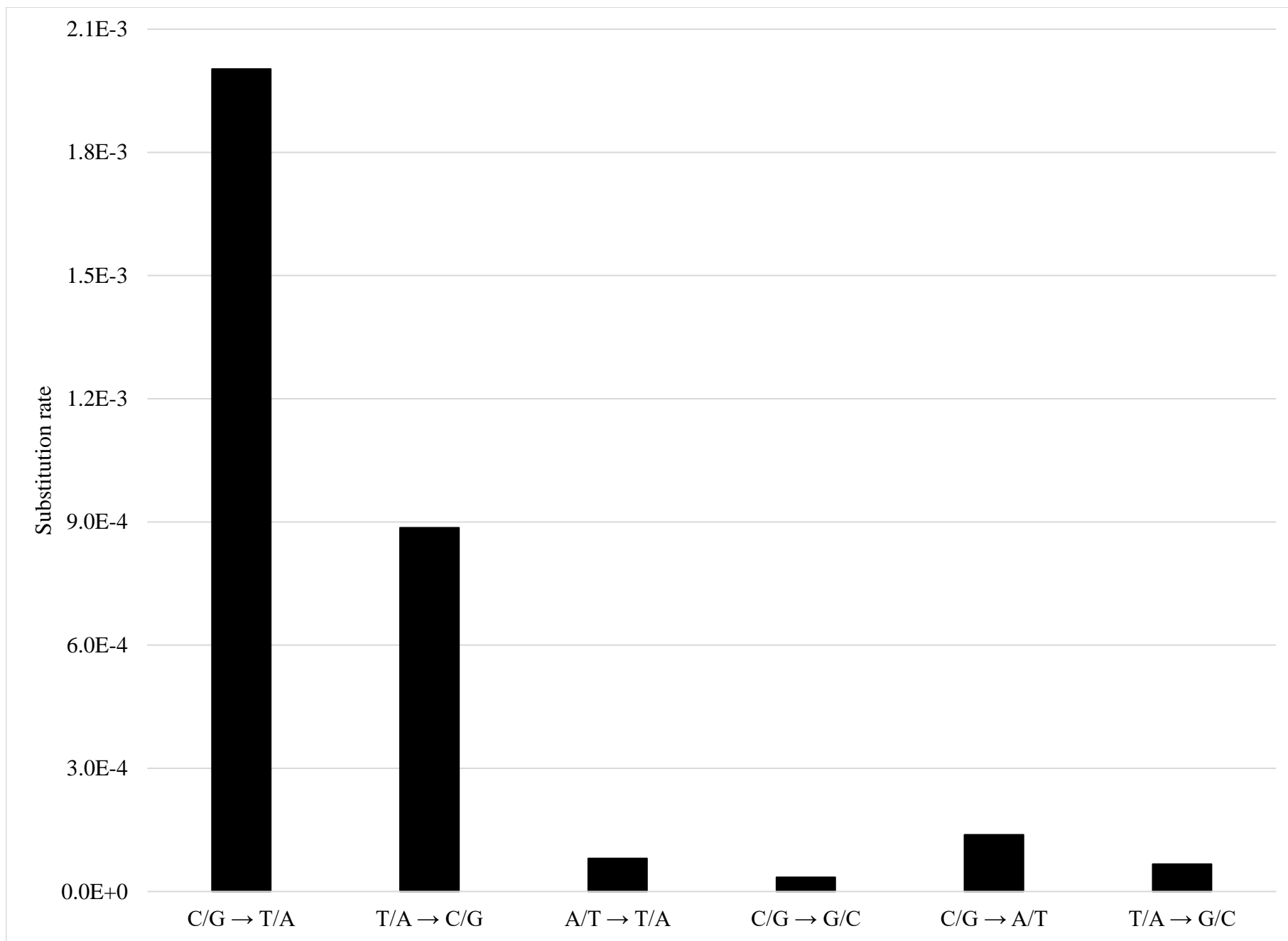

Supplement: Supplemental Information 5 [file peerj-09-10420-s005.pdf]

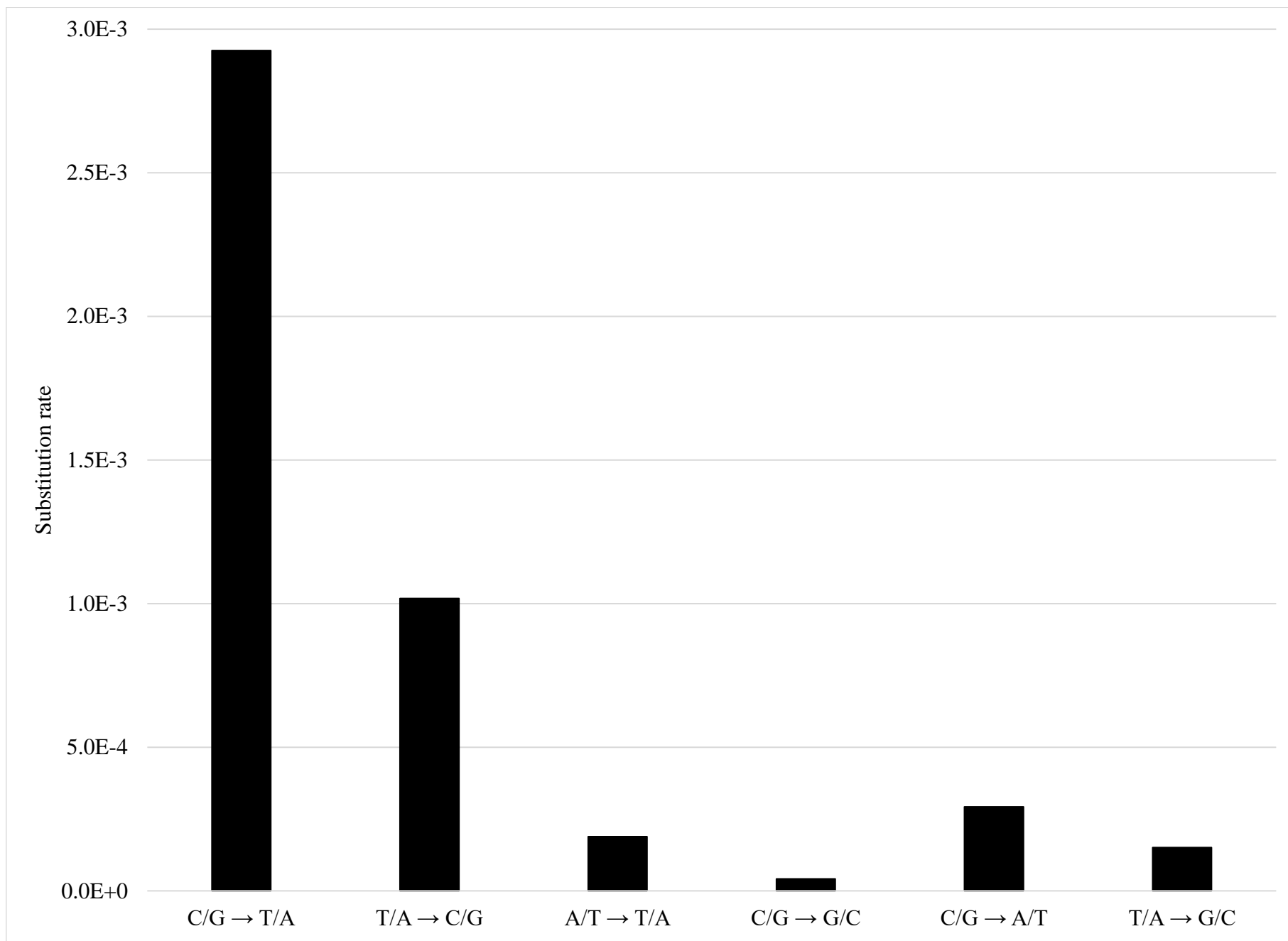

Supplement: Supplemental Information 6 [file peerj-09-10420-s006.pdf]

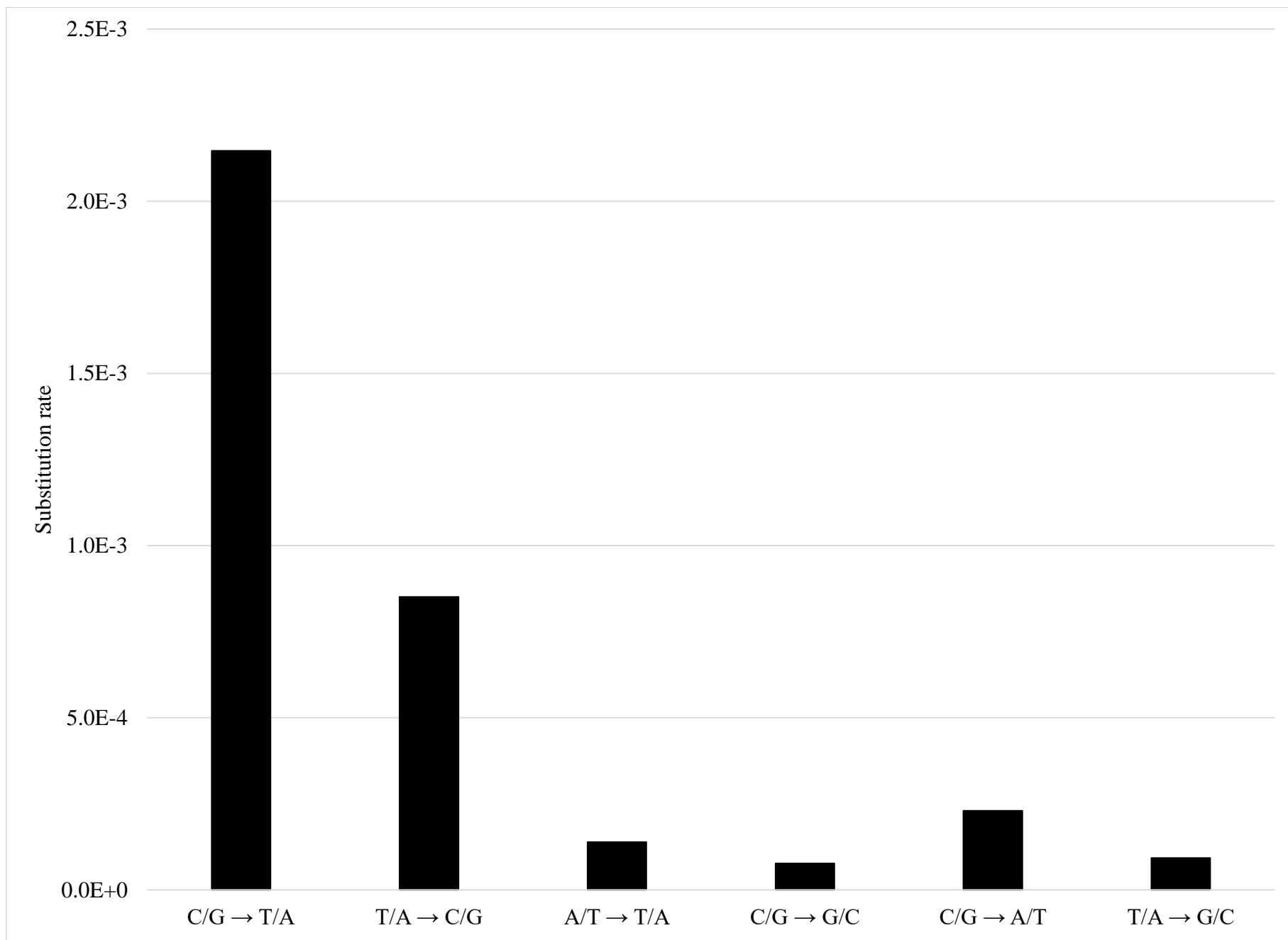

Supplement: Supplemental Information 7 [file peerj-09-10420-s007.pdf]

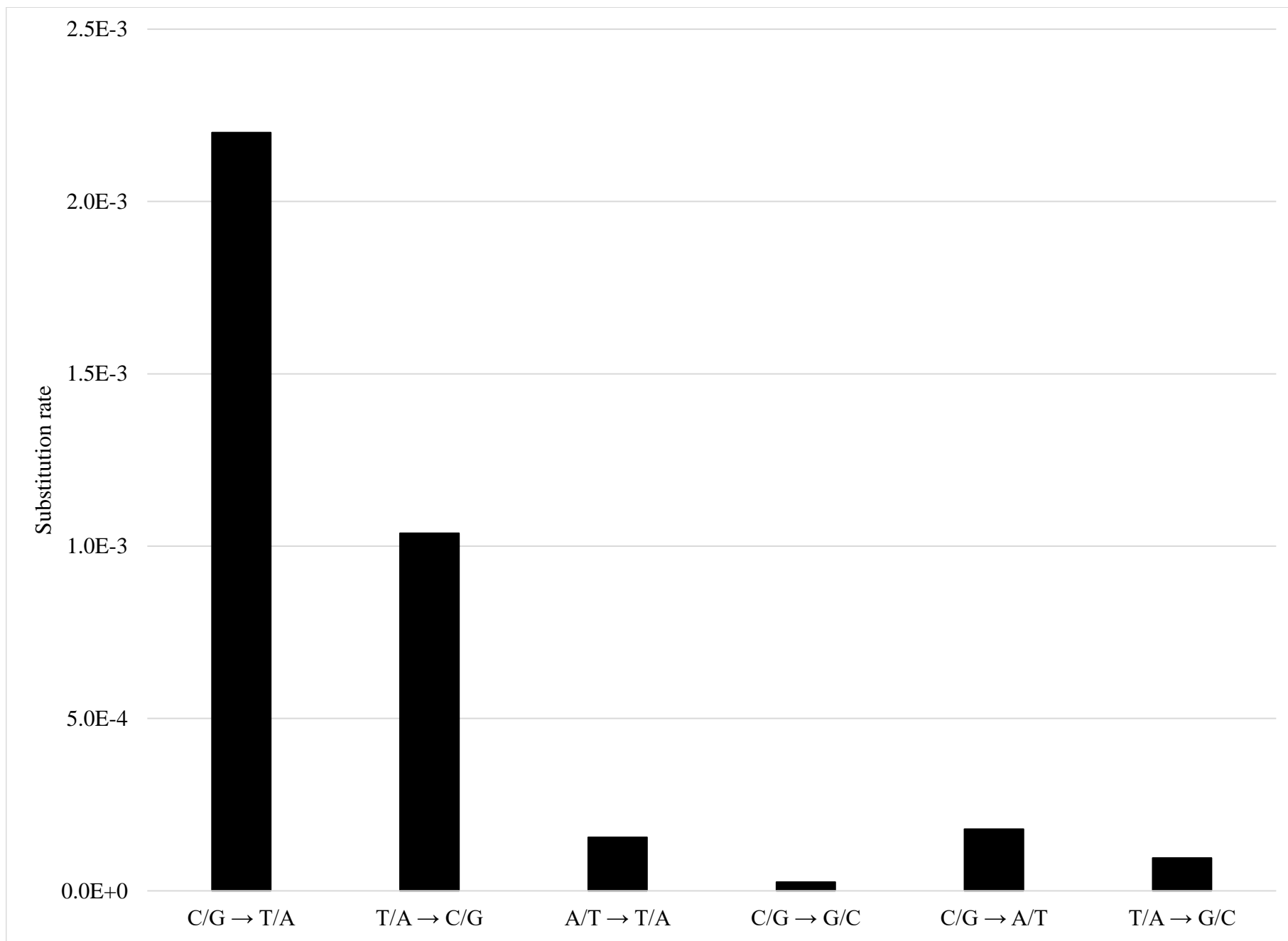

Supplement: Supplemental Information 8 [file peerj-09-10420-s008.pdf]

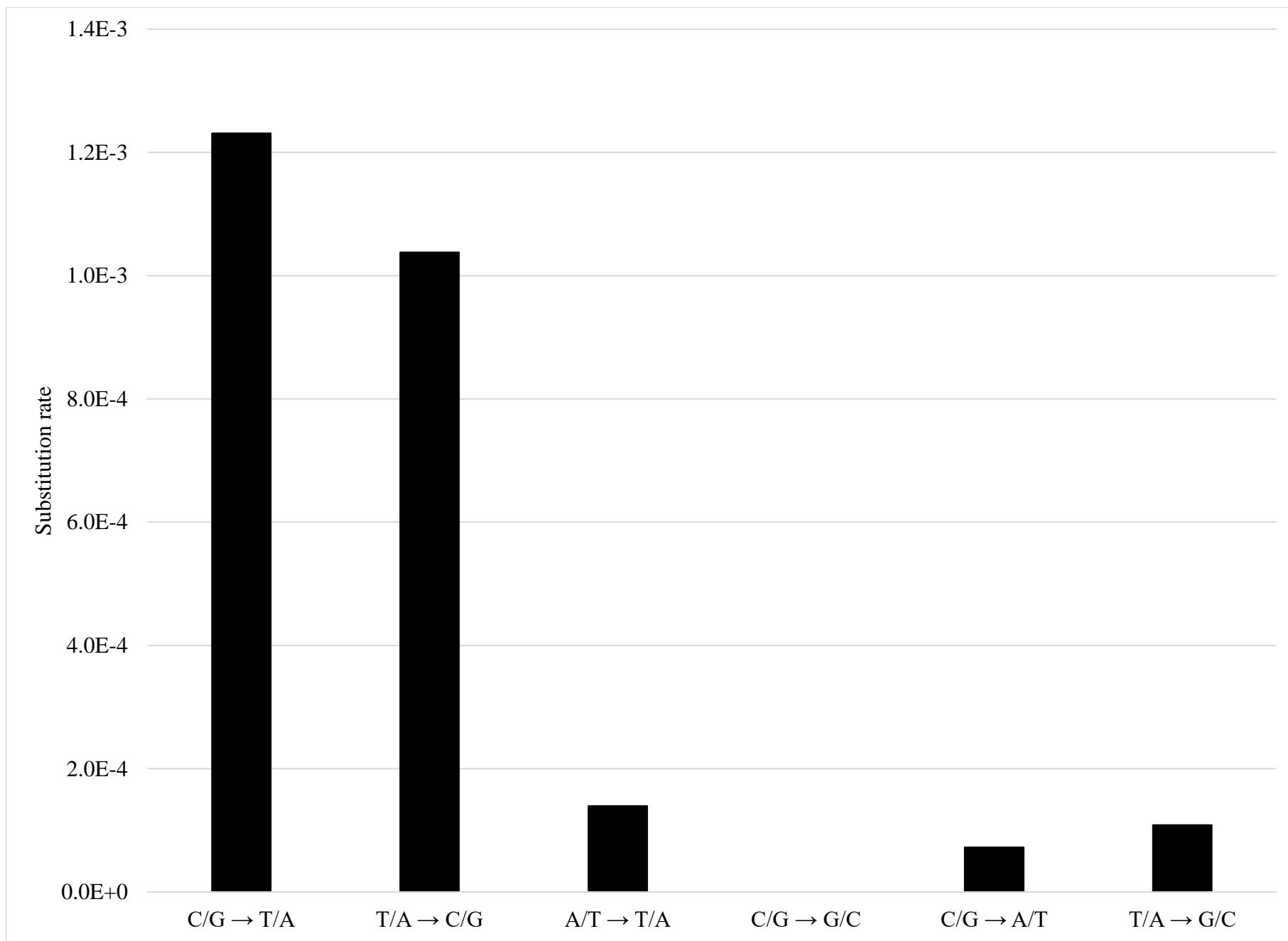

Supplement: Supplemental Information 9 [file peerj-09-10420-s009.pdf]

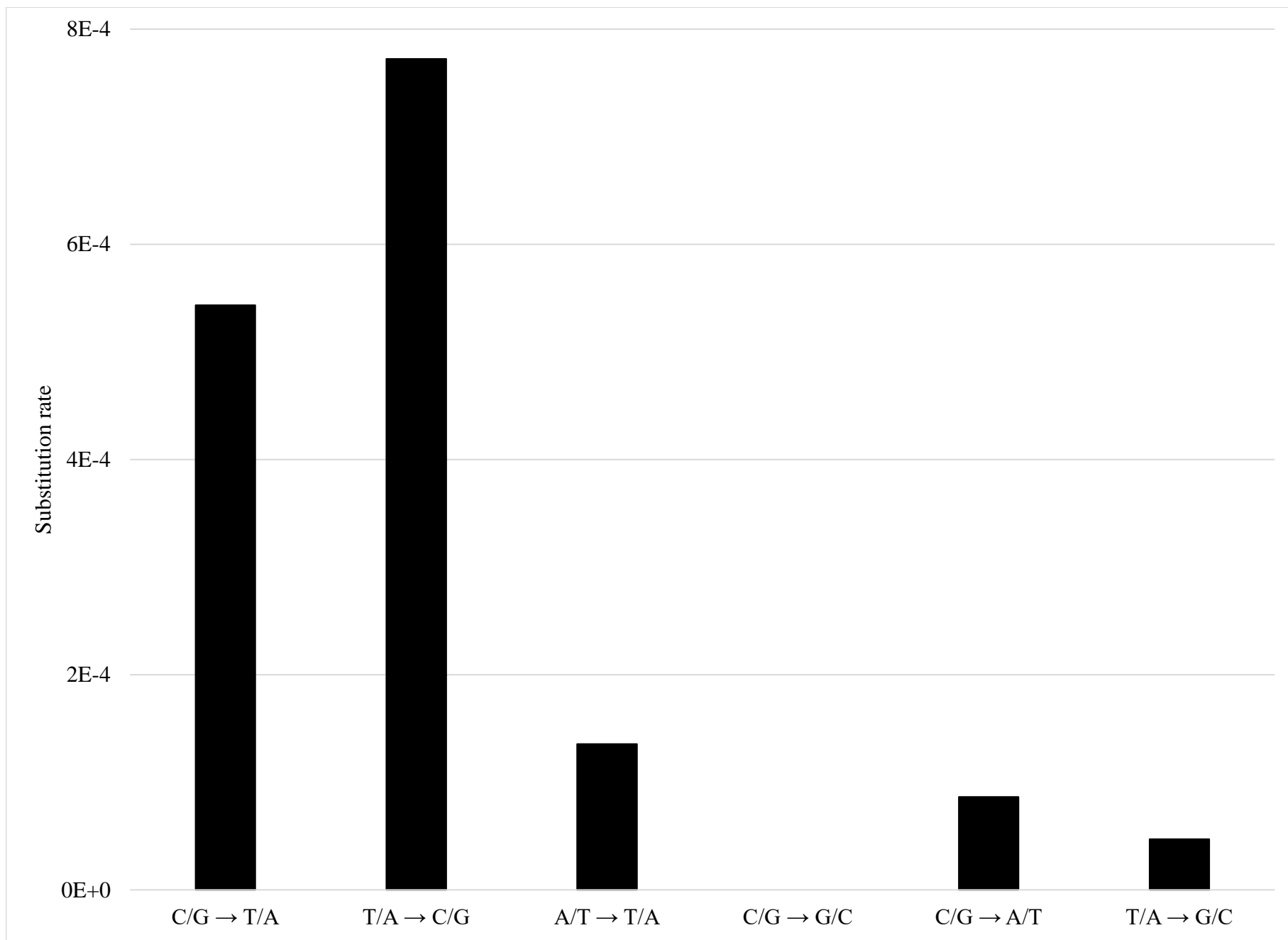

Supplement: Supplemental Information 10 [file peerj-09-10420-s010.pdf]

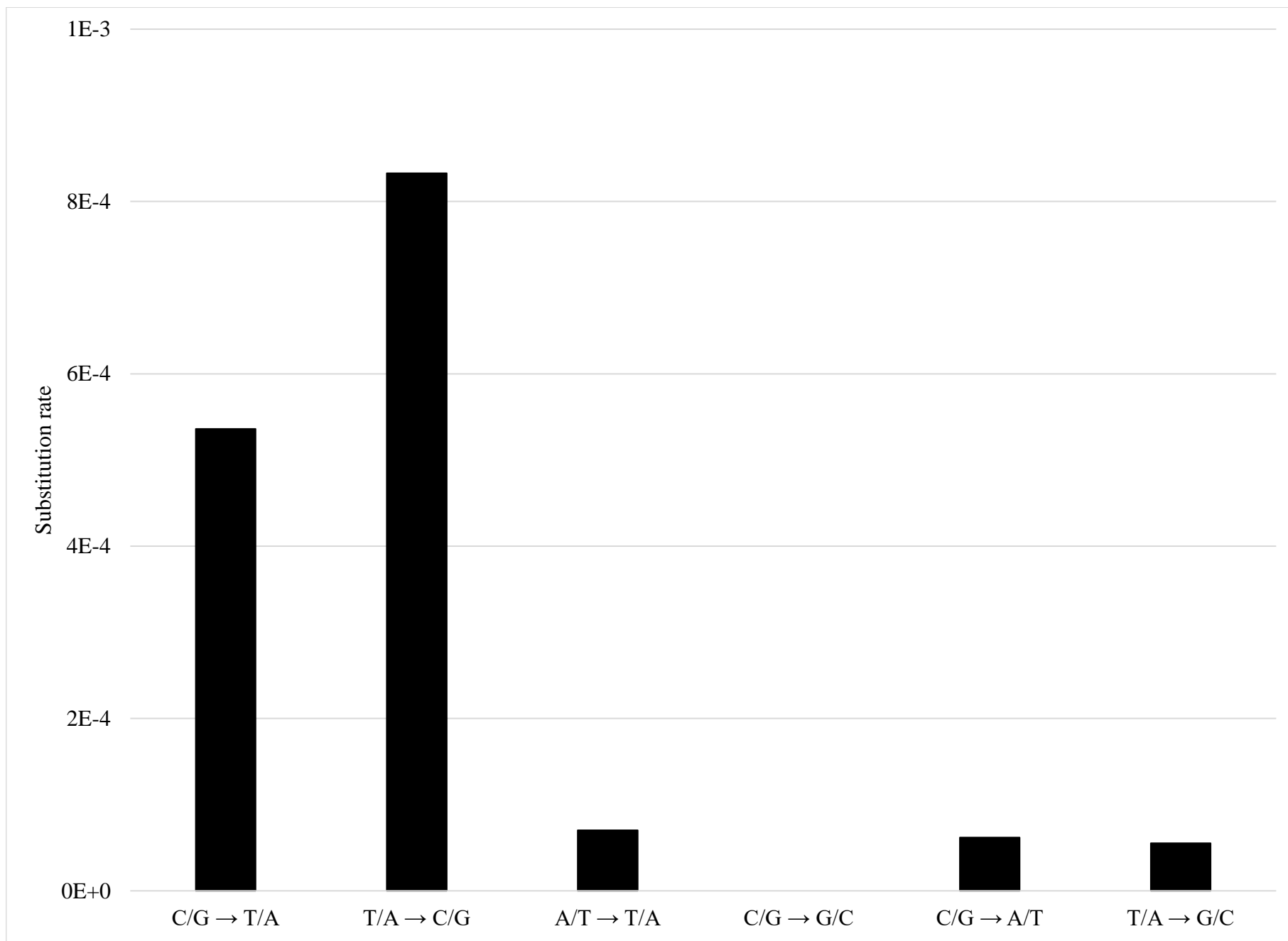

Supplement: Supplemental Information 11 [file peerj-09-10420-s011.pdf]

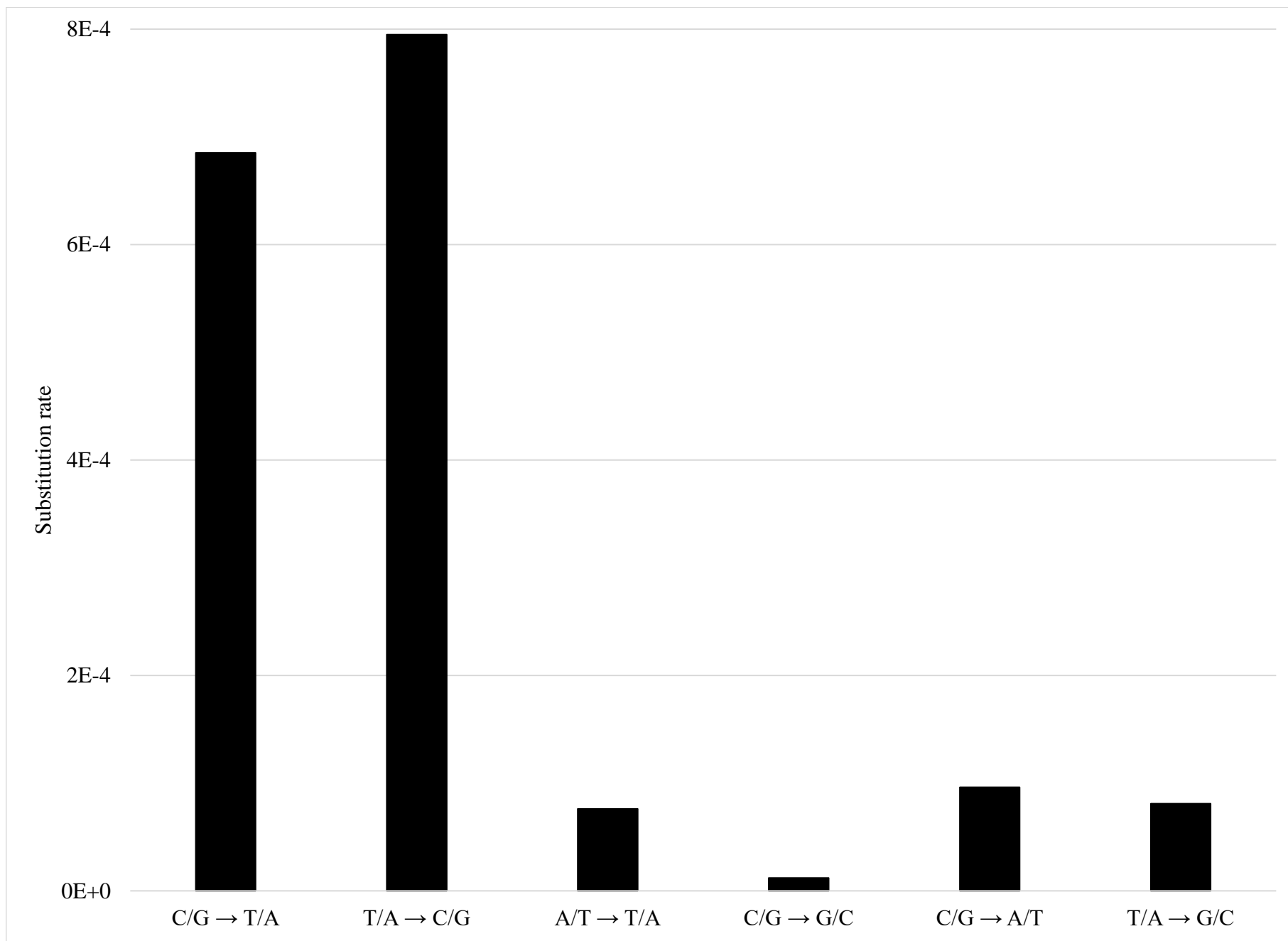

Supplement: Supplemental Information 12 [file peerj-09-10420-s012.pdf]

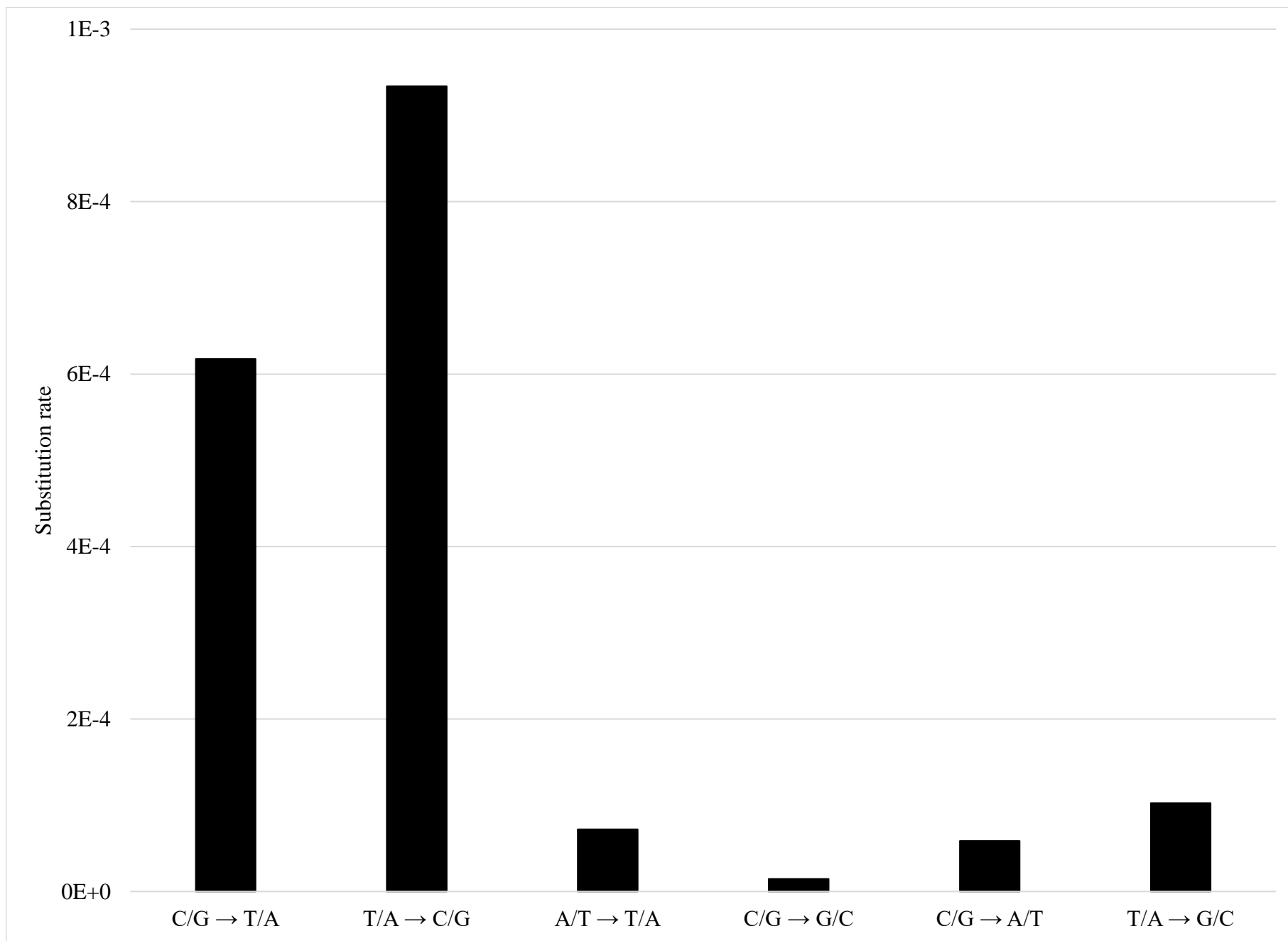

Supplement: Supplemental Information 13 [file peerj-09-10420-s013.pdf]

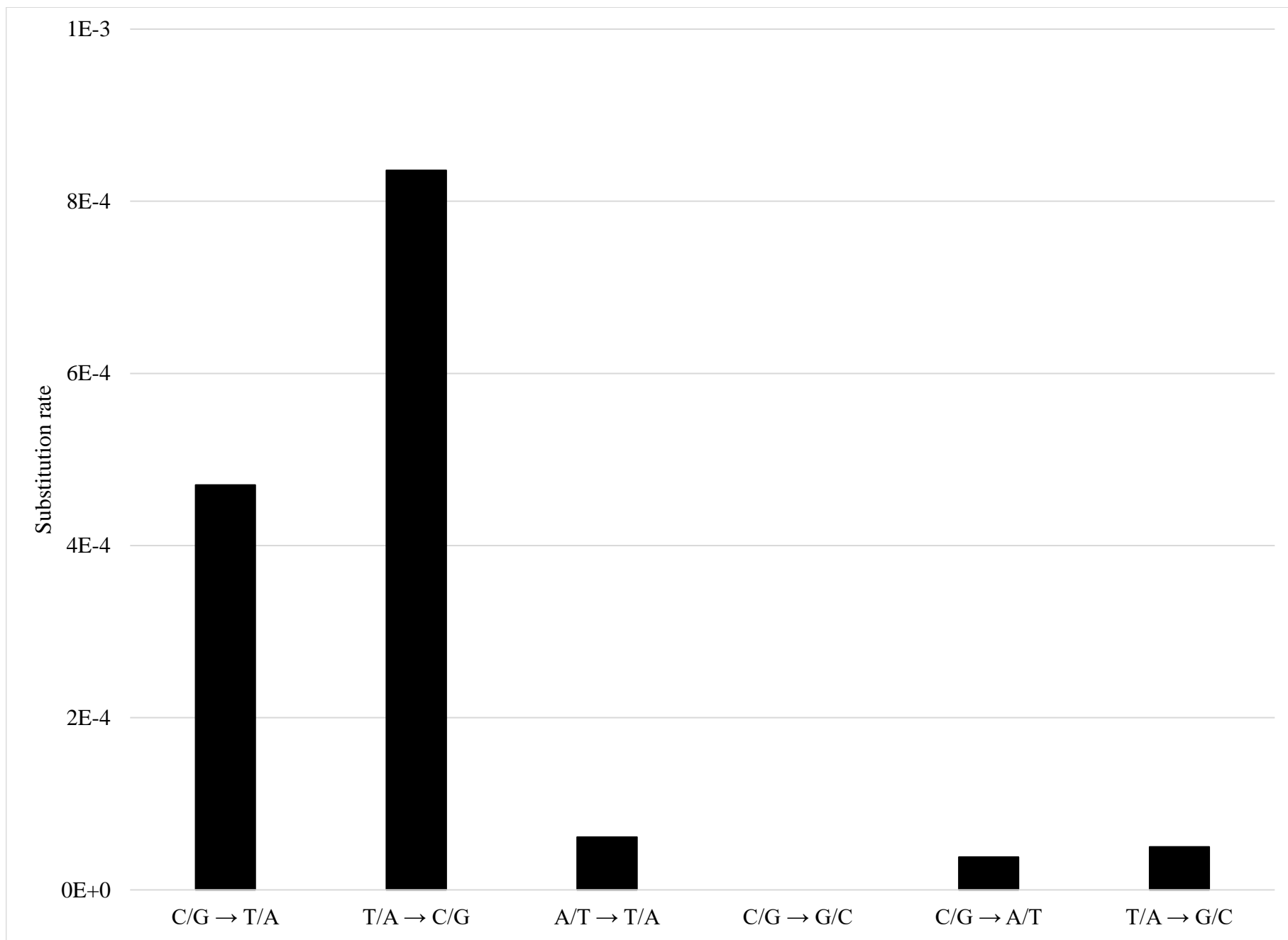

Supplement: Supplemental Information 14 [file peerj-09-10420-s014.pdf]

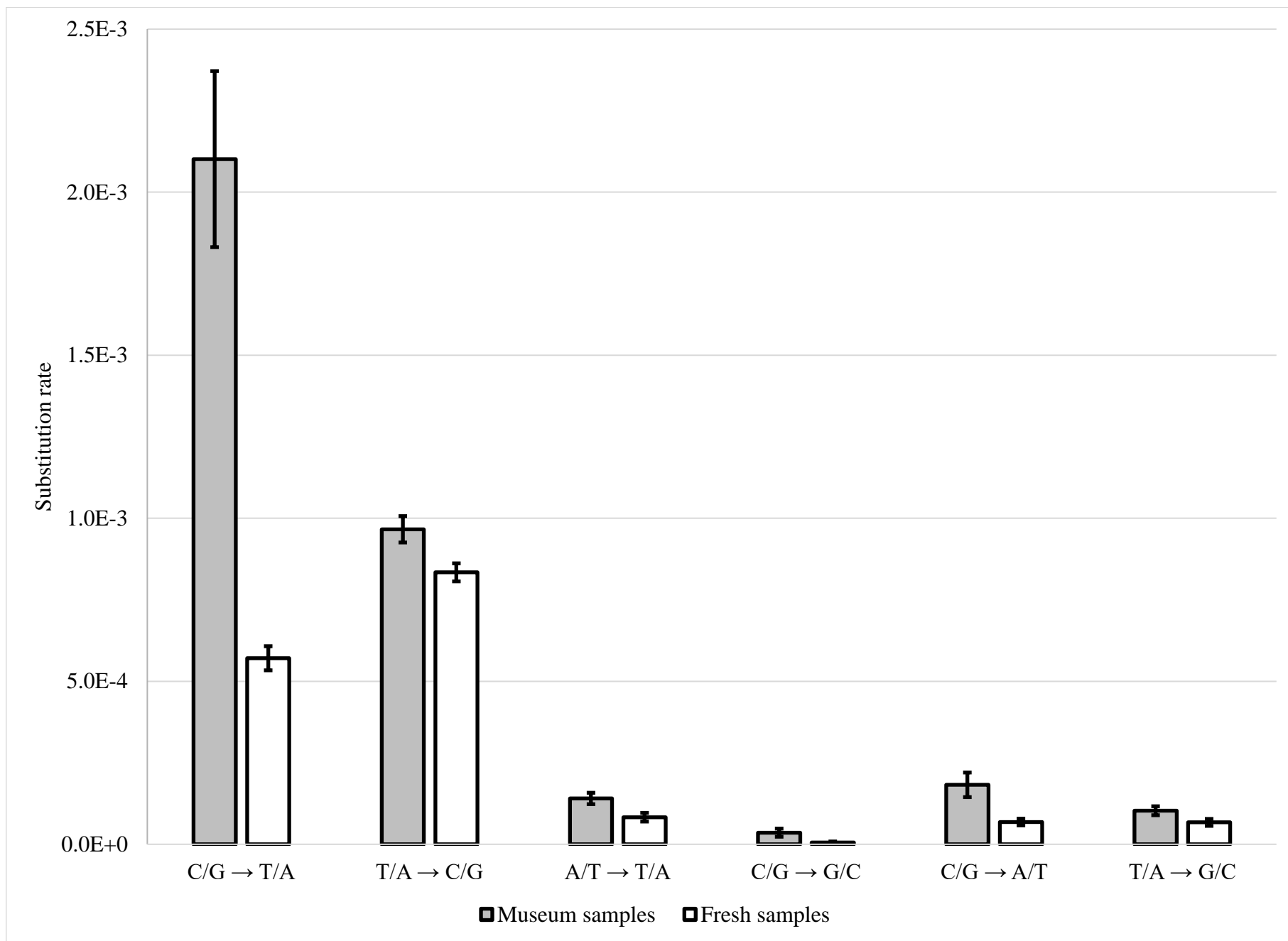

Supplement: Supplemental Information 15 — Substitution rates for each type of transition and transversion; bars show the standard error. [file peerj-09-10420-s015.pdf]
